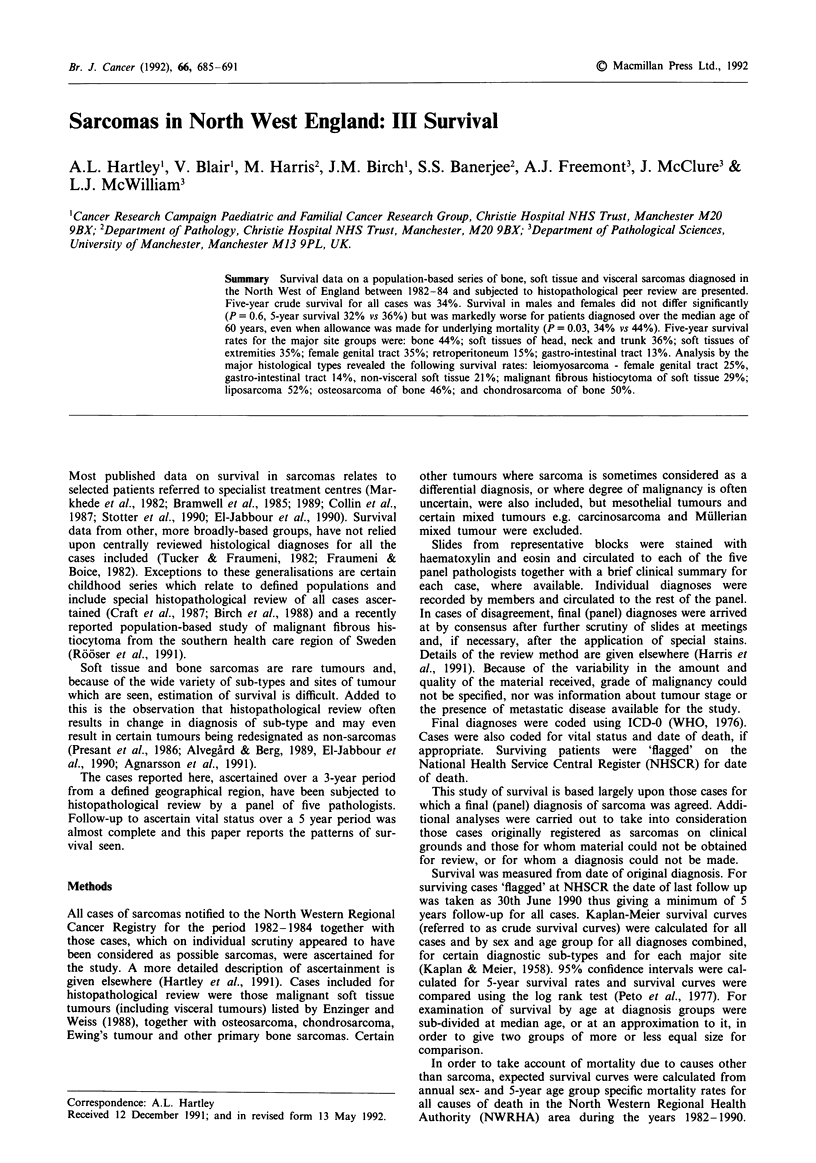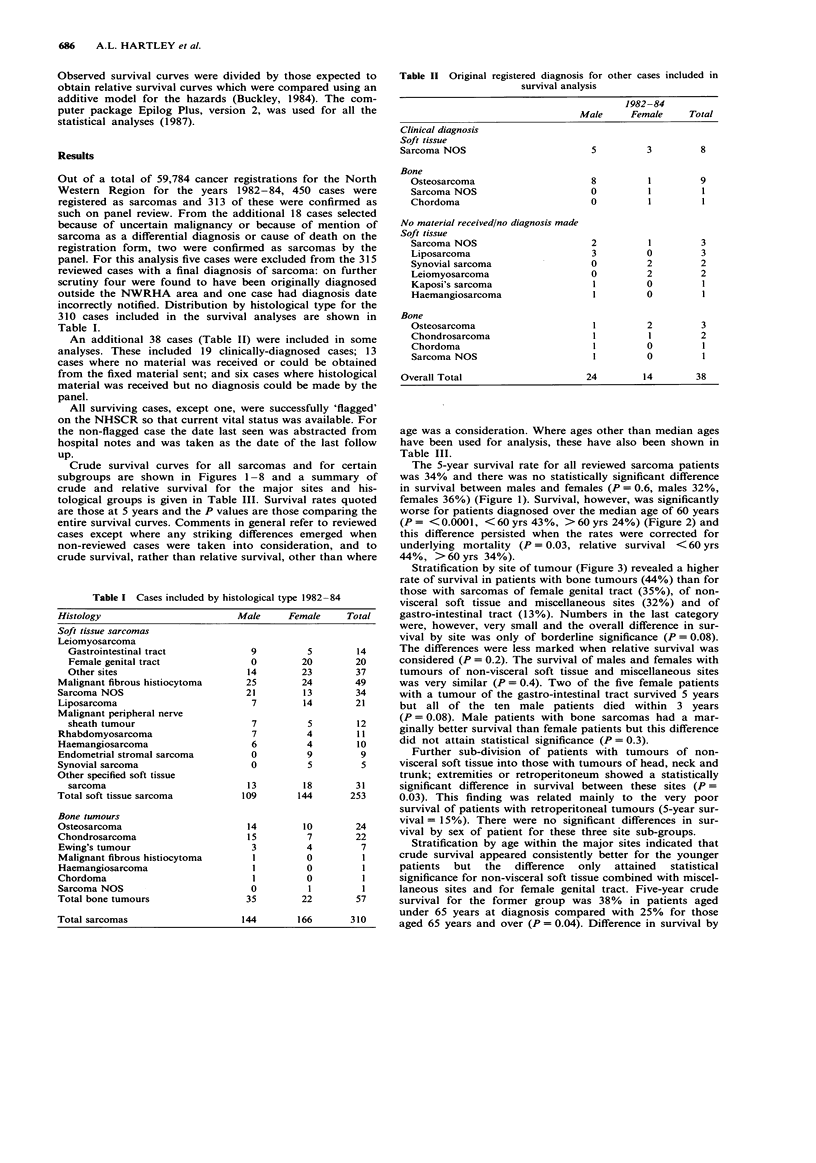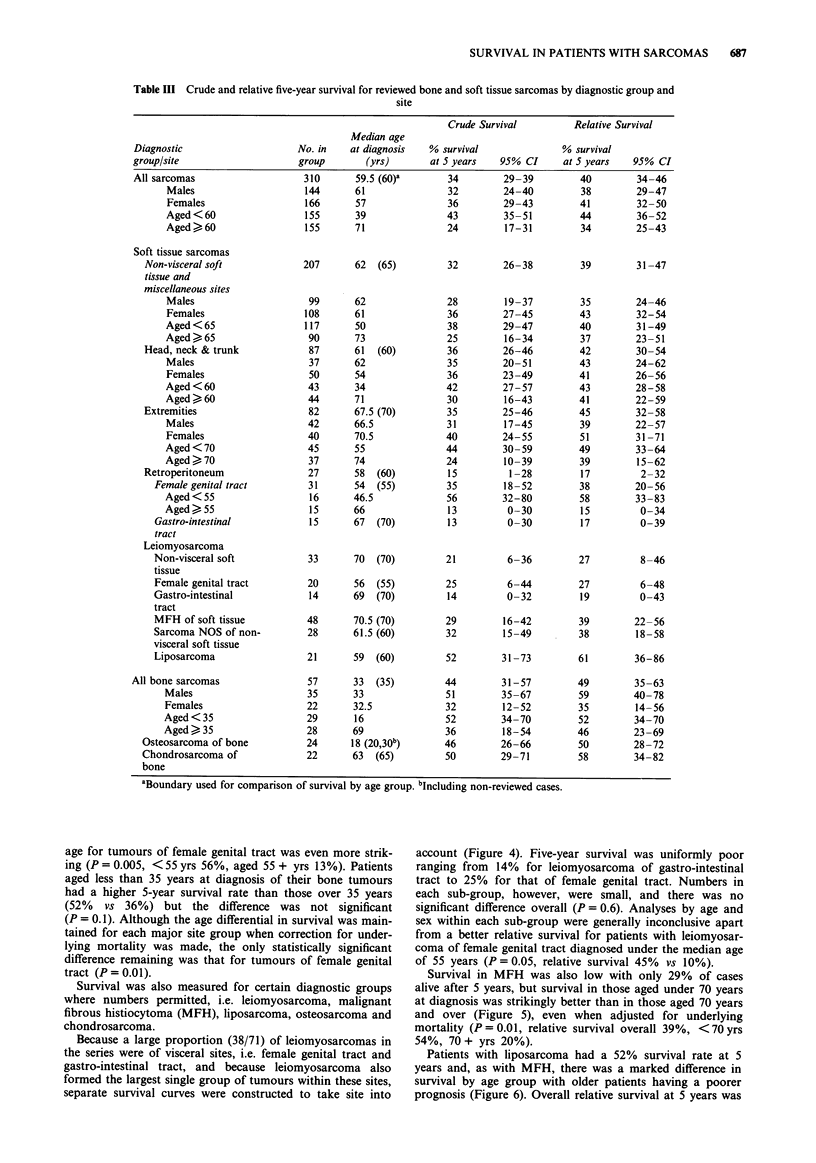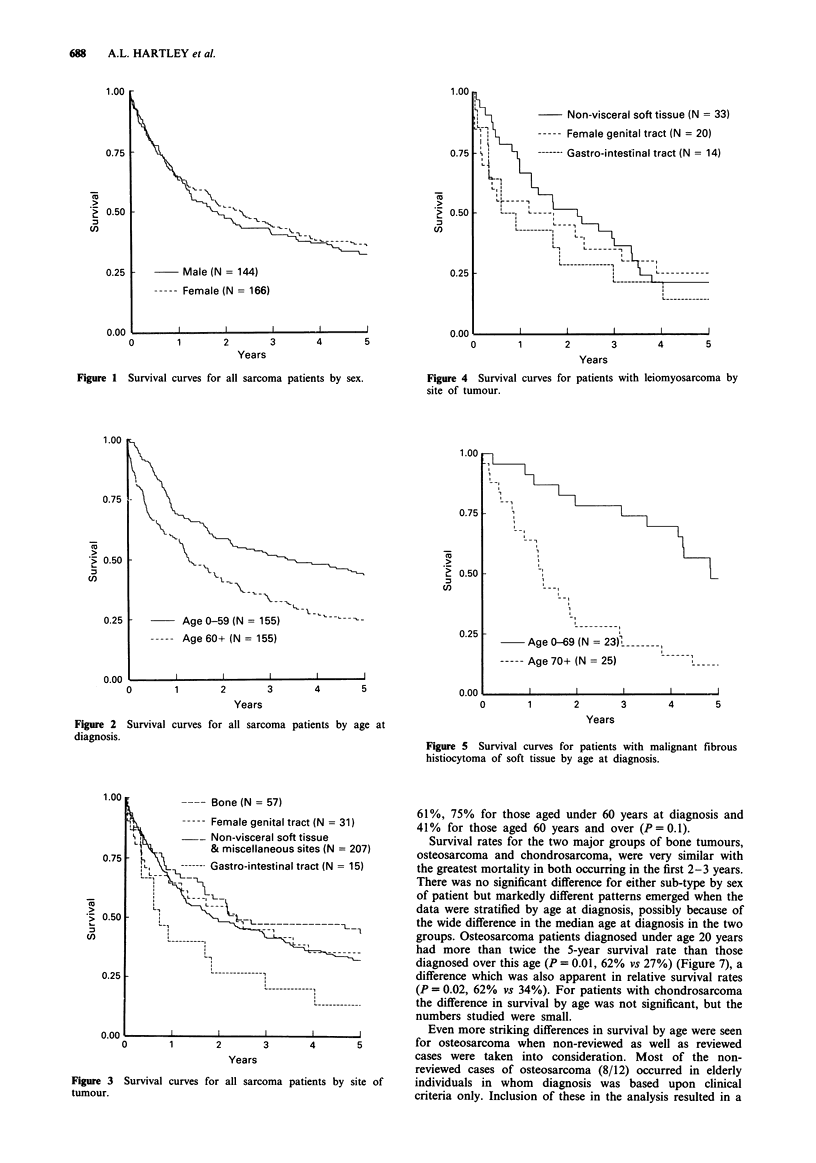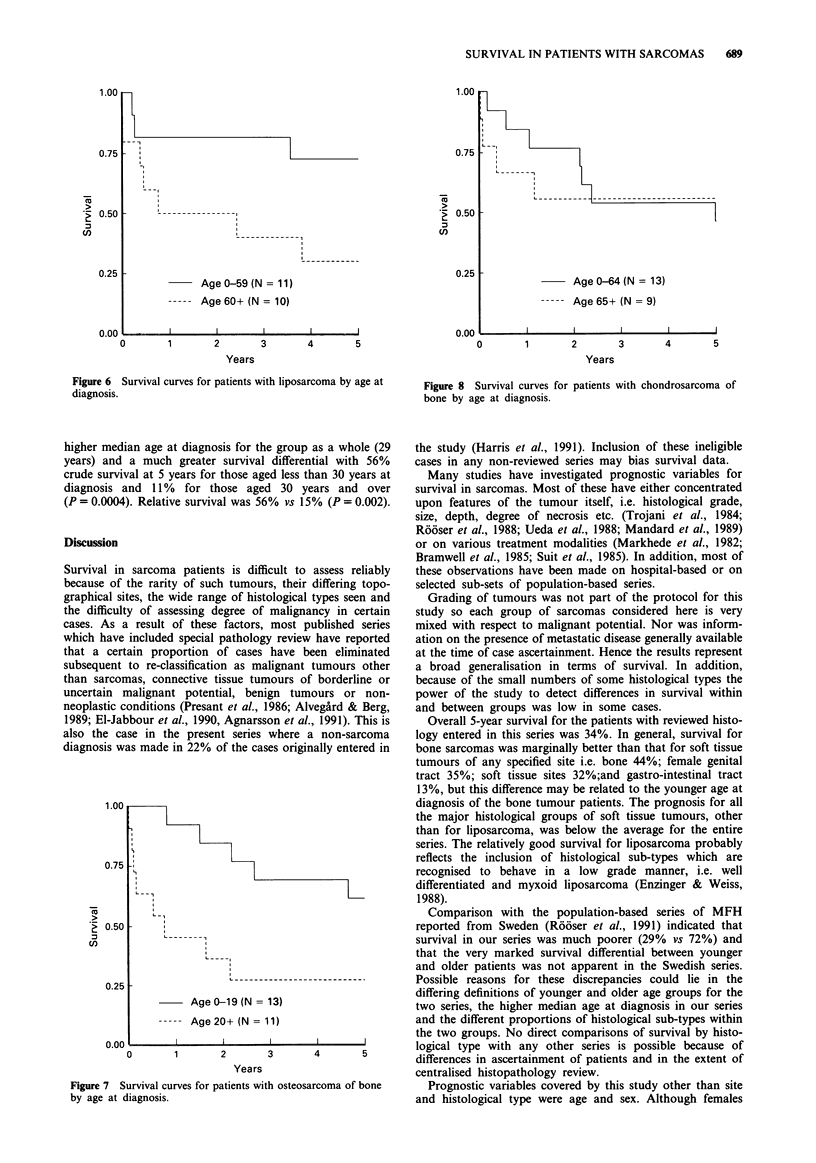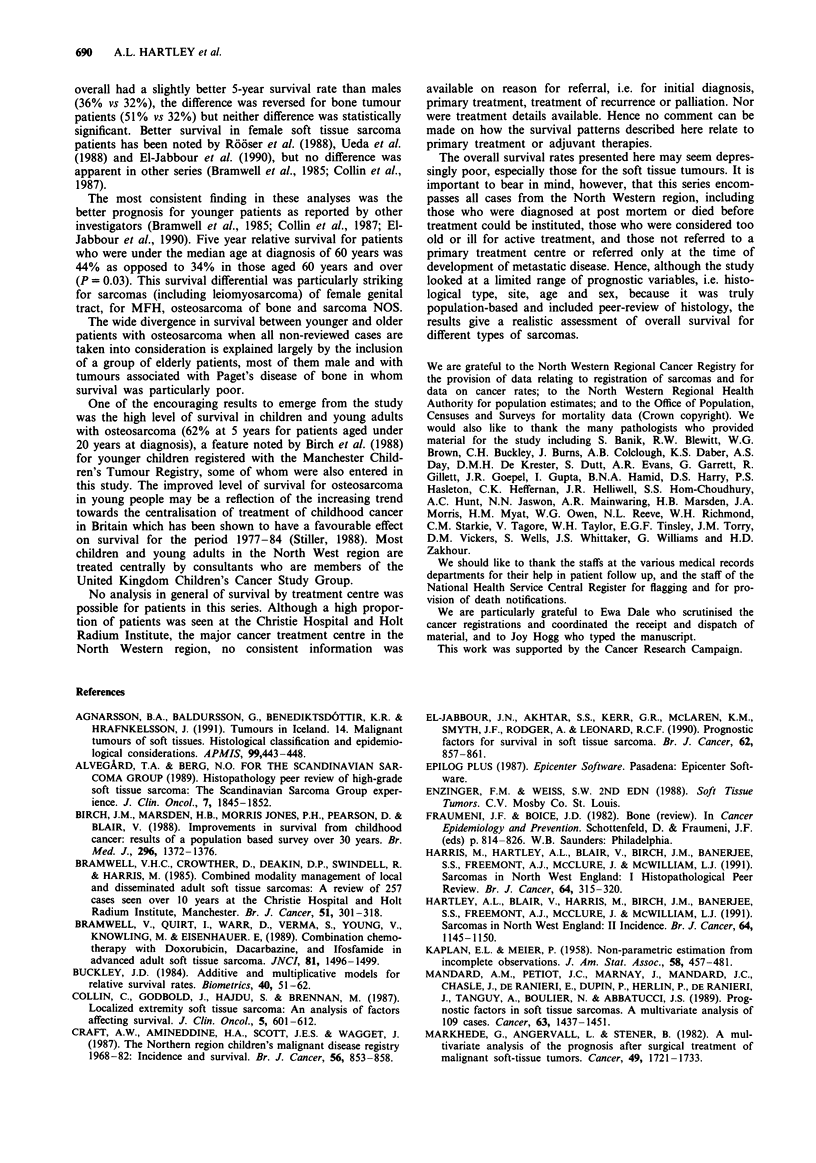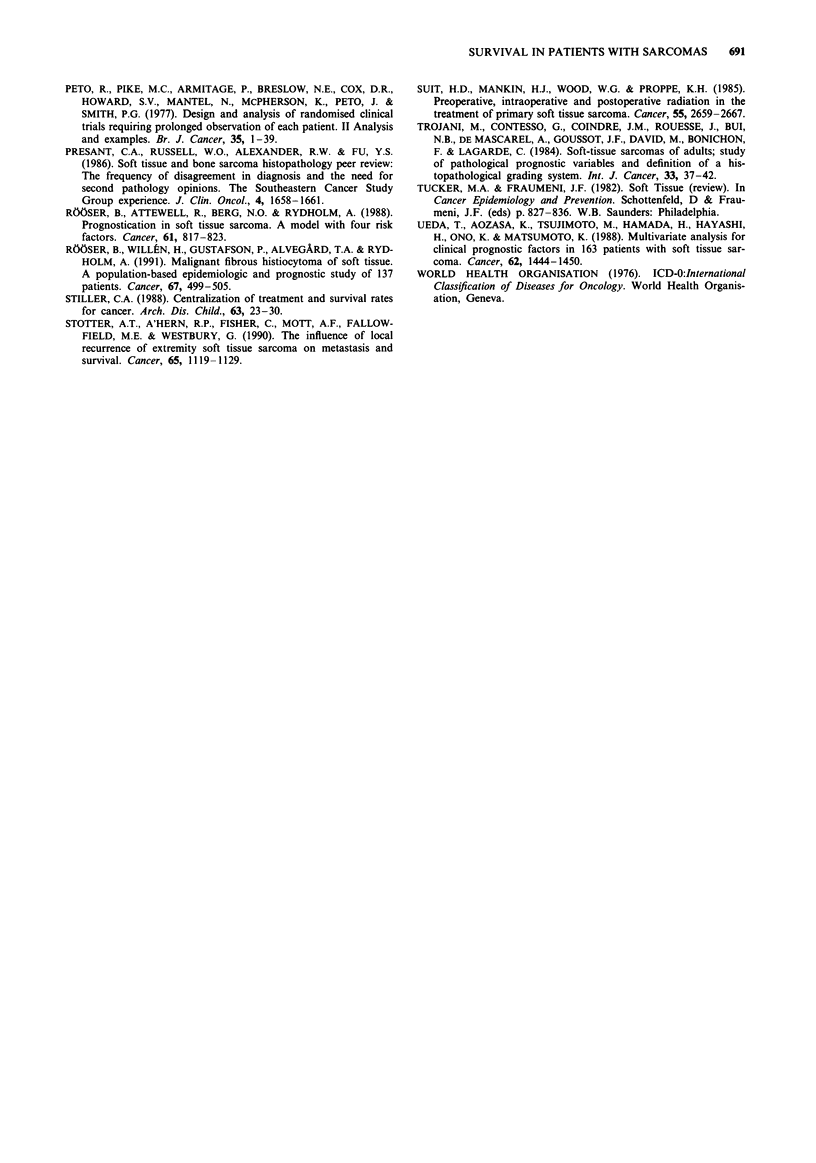# Sarcomas in north west England: III. Survival.

**DOI:** 10.1038/bjc.1992.338

**Published:** 1992-10

**Authors:** A. L. Hartley, V. Blair, M. Harris, J. M. Birch, S. S. Banerjee, A. J. Freemont, J. McClure, L. J. McWilliam

**Affiliations:** Cancer Research Campaign Paediatric and Familial Cancer Research Group, Christie Hospital NHS Trust, Manchester, UK.

## Abstract

Survival data on a population-based series of bone, soft tissue and visceral sarcomas diagnosed in the North West of England between 1982-84 and subjected to histopathological peer review are presented. Five-year crude survival for all cases was 34%. Survival in males and females did not differ significantly (P = 0.6, 5-year survival 32% vs 36%) but was markedly worse for patients diagnosed over the median age of 60 years, even when allowance was made for underlying mortality (P = 0.03, 34% vs 44%). Five-year survival rates for the major site groups were: bone 44%; soft tissues of head, neck and trunk 36%; soft tissues of extremities 35%; female genital tract 35%; retroperitoneum 15%; gastro-intestinal tract 13%. Analysis by the major histological types revealed the following survival rates: leiomyosarcoma--female genital tract 25%, gastro-intestinal tract 14%, non-visceral soft tissue 21%; malignant fibrous histiocytoma of soft tissue 29%; liposarcoma 52%; osteosarcoma of bone 46%; and chondrosarcoma of bone 50%.


Br. J. Cancer (1992), 66, 685-691 © Macmillan Press Ltd., 1992

### Sarcomas in North West England: III Survival

A.L. Hartley[1], V. Blair[1], M. Harris[2], J.M. Birch[1], S.S. Banerjee[2], A.J. Freemont[3], J. McClure[3] & L.J. McWilliam[3]

[1]*Cancer Research Campaign Paediatric and Familial Cancer Research Group, Christie Hospital NHS Trust, Manchester M20 9BX;* [2]*Department of Pathology, Christie Hospital NHS Trust, Manchester, M20 9BX;* [3]*Department of Pathological Sciences, University of Manchester, Manchester M13 9PL, UK.*

**Summary** Survival data on a population-based series of bone, soft tissue and visceral sarcomas diagnosed in the North West of England between 1982-84 and subjected to histopathological peer review are presented. Five-year crude survival for all cases was 34%. Survival in males and females did not differ significantly ($P = 0.6$, 5-year survival 32% *vs* 36%) but was markedly worse for patients diagnosed over the median age of 60 years, even when allowance was made for underlying mortality ($P = 0.03$, 34% *vs* 44%). Five-year survival rates for the major site groups were: bone 44%; soft tissues of head, neck and trunk 36%; soft tissues of extremities 35%; female genital tract 35%; retroperitoneum 15%; gastro-intestinal tract 13%. Analysis by the major histological types revealed the following survival rates: leiomyosarcoma - female genital tract 25%, gastro-intestinal tract 14%, non-visceral soft tissue 21%; malignant fibrous histiocytoma of soft tissue 29%; liposarcoma 52%; osteosarcoma of bone 46%; and chondrosarcoma of bone 50%.

Most published data on survival in sarcomas relates to selected patients referred to specialist treatment centres (Markhede *et al.*, 1982; Bramwell *et al.*, 1985; 1989; Collin *et al.*, 1987; Stotter *et al.*, 1990; El-Jabbour *et al.*, 1990). Survival data from other, more broadly-based groups, have not relied upon centrally reviewed histological diagnoses for all the cases included (Tucker & Fraumeni, 1982; Fraumeni & Boice, 1982). Exceptions to these generalisations are certain childhood series which relate to defined populations and include special histopathological review of all cases ascertained (Craft *et al.*, 1987; Birch *et al.*, 1988) and a recently reported population-based study of malignant fibrous histiocytoma from the southern health care region of Sweden (Rööser *et al.*, 1991).

Soft tissue and bone sarcomas are rare tumours and, because of the wide variety of sub-types and sites of tumour which are seen, estimation of survival is difficult. Added to this is the observation that histopathological review often results in change in diagnosis of sub-type and may even result in certain tumours being redesignated as non-sarcomas (Presant *et al.*, 1986; Alvegård & Berg, 1989, El-Jabbour *et al.*, 1990; Agnarsson *et al.*, 1991).

The cases reported here, ascertained over a 3-year period from a defined geographical region, have been subjected to histopathological review by a panel of five pathologists. Follow-up to ascertain vital status over a 5 year period was almost complete and this paper reports the patterns of survival seen.

### Methods

All cases of sarcomas notified to the North Western Regional Cancer Registry for the period 1982-1984 together with those cases, which on individual scrutiny appeared to have been considered as possible sarcomas, were ascertained for the study. A more detailed description of ascertainment is given elsewhere (Hartley *et al.*, 1991). Cases included for histopathological review were those malignant soft tissue tumours (including visceral tumours) listed by Enzinger and Weiss (1988), together with osteosarcoma, chondrosarcoma, Ewing's tumour and other primary bone sarcomas. Certain other tumours where sarcoma is sometimes considered as a differential diagnosis, or where degree of malignancy is often uncertain, were also included, but mesothelial tumours and certain mixed tumours e.g. carcinosarcoma and Müllerian mixed tumour were excluded.

Slides from representative blocks were stained with haematoxylin and eosin and circulated to each of the five panel pathologists together with a brief clinical summary for each case, where available. Individual diagnoses were recorded by members and circulated to the rest of the panel. In cases of disagreement, final (panel) diagnoses were arrived at by consensus after further scrutiny of slides at meetings and, if necessary, after the application of special stains. Details of the review method are given elsewhere (Harris *et al.*, 1991). Because of the variability in the amount and quality of the material received, grade of malignancy could not be specified, nor was information about tumour stage or the presence of metastatic disease available for the study.

Final diagnoses were coded using ICD-0 (WHO, 1976). Cases were also coded for vital status and date of death, if appropriate. Surviving patients were 'flagged' on the National Health Service Central Register (NHSCR) for date of death.

This study of survival is based largely upon those cases for which a final (panel) diagnosis of sarcoma was agreed. Additional analyses were carried out to take into consideration those cases originally registered as sarcomas on clinical grounds and those for whom material could not be obtained for review, or for whom a diagnosis could not be made.

Survival was measured from date of original diagnosis. For surviving cases 'flagged' at NHSCR the date of last follow up was taken as 30th June 1990 thus giving a minimum of 5 years follow-up for all cases. Kaplan-Meier survival curves (referred to as crude survival curves) were calculated for all cases and by sex and age group for all diagnoses combined, for certain diagnostic sub-types and for each major site (Kaplan & Meier, 1958). 95% confidence intervals were calculated for 5-year survival rates and survival curves were compared using the log rank test (Peto *et al.*, 1977). For examination of survival by age at diagnosis groups were sub-divided at median age, or at an approximation to it, in order to give two groups of more or less equal size for comparison.

In order to take account of mortality due to causes other than sarcoma, expected survival curves were calculated from annual sex- and 5-year age group specific mortality rates for all causes of death in the North Western Regional Health Authority (NWRHA) area during the years 1982-1990.

Correspondence: A.L. Hartley
Received 12 December 1991; and in revised form 13 May 1992.

Observed survival curves were divided by those expected to obtain relative survival curves which were compared using an additive model for the hazards (Buckley, 1984). The computer package Epilog Plus, version 2, was used for all the statistical analyses (1987).

### Results

Out of a total of 59,784 cancer registrations for the North Western Region for the years 1982–84, 450 cases were registered as sarcomas and 313 of these were confirmed as such on panel review. From the additional 18 cases selected because of uncertain malignancy or because of mention of sarcoma as a differential diagnosis or cause of death on the registration form, two were confirmed as sarcomas by the panel. For this analysis five cases were excluded from the 315 reviewed cases with a final diagnosis of sarcoma: on further scrutiny four were found to have been originally diagnosed outside the NWRHA area and one case had diagnosis date incorrectly notified. Distribution by histological type for the 310 cases included in the survival analyses are shown in Table I.

An additional 38 cases (Table II) were included in some analyses. These included 19 clinically-diagnosed cases; 13 cases where no material was received or could be obtained from the fixed material sent; and six cases where histological material was received but no diagnosis could be made by the panel.

All surviving cases, except one, were successfully 'flagged' on the NHSCR so that current vital status was available. For the non-flagged case the date last seen was abstracted from hospital notes and was taken as the date of the last follow up.

Crude survival curves for all sarcomas and for certain subgroups are shown in Figures 1–8 and a summary of crude and relative survival for the major sites and histological groups is given in Table III. Survival rates quoted are those at 5 years and the $P$ values are those comparing the entire survival curves. Comments in general refer to reviewed cases except where any striking differences emerged when non-reviewed cases were taken into consideration, and to crude survival, rather than relative survival, other than where age was a consideration. Where ages other than median ages have been used for analysis, these have also been shown in Table III.

**Table I** Cases included by histological type 1982–84

| *Histology* | *Male* | *Female* | *Total* |
|---|---|---|---|
| *Soft tissue sarcomas* | | | |
| Leiomyosarcoma | | | |
| Gastrointestinal tract | 9 | 5 | 14 |
| Female genital tract | 0 | 20 | 20 |
| Other sites | 14 | 23 | 37 |
| Malignant fibrous histiocytoma | 25 | 24 | 49 |
| Sarcoma NOS | 21 | 13 | 34 |
| Liposarcoma | 7 | 14 | 21 |
| Malignant peripheral nerve sheath tumour | 7 | 5 | 12 |
| Rhabdomyosarcoma | 7 | 4 | 11 |
| Haemangiosarcoma | 6 | 4 | 10 |
| Endometrial stromal sarcoma | 0 | 9 | 9 |
| Synovial sarcoma | 0 | 5 | 5 |
| Other specified soft tissue sarcoma | 13 | 18 | 31 |
| Total soft tissue sarcoma | 109 | 144 | 253 |
| *Bone tumours* | | | |
| Osteosarcoma | 14 | 10 | 24 |
| Chondrosarcoma | 15 | 7 | 22 |
| Ewing's tumour | 3 | 4 | 7 |
| Malignant fibrous histiocytoma | 1 | 0 | 1 |
| Haemangiosarcoma | 1 | 0 | 1 |
| Chordoma | 1 | 0 | 1 |
| Sarcoma NOS | 0 | 1 | 1 |
| Total bone tumours | 35 | 22 | 57 |
| Total sarcomas | 144 | 166 | 310 |

**Table II** Original registered diagnosis for other cases included in survival analysis

| | | *1982–84* | |
|---|---|---|---|
| | *Male* | *Female* | *Total* |
| *Clinical diagnosis* | | | |
| *Soft tissue* | | | |
| Sarcoma NOS | 5 | 3 | 8 |
| *Bone* | | | |
| Osteosarcoma | 8 | 1 | 9 |
| Sarcoma NOS | 0 | 1 | 1 |
| Chordoma | 0 | 1 | 1 |
| *No material received/no diagnosis made* | | | |
| *Soft tissue* | | | |
| Sarcoma NOS | 2 | 1 | 3 |
| Liposarcoma | 3 | 0 | 3 |
| Synovial sarcoma | 0 | 2 | 2 |
| Leiomyosarcoma | 0 | 2 | 2 |
| Kaposi's sarcoma | 1 | 0 | 1 |
| Haemangiosarcoma | 1 | 0 | 1 |
| *Bone* | | | |
| Osteosarcoma | 1 | 2 | 3 |
| Chondrosarcoma | 1 | 1 | 2 |
| Chordoma | 1 | 0 | 1 |
| Sarcoma NOS | 1 | 0 | 1 |
| Overall Total | 24 | 14 | 38 |

The 5-year survival rate for all reviewed sarcoma patients was 34% and there was no statistically significant difference in survival between males and females ($P = 0.6$, males 32%, females 36%) (Figure 1). Survival, however, was significantly worse for patients diagnosed over the median age of 60 years ($P = < 0.0001$, $< 60$ yrs 43%, $> 60$ yrs 24%) (Figure 2) and this difference persisted when the rates were corrected for underlying mortality ($P = 0.03$, relative survival $< 60$ yrs 44%, $> 60$ yrs 34%).

Stratification by site of tumour (Figure 3) revealed a higher rate of survival in patients with bone tumours (44%) than for those with sarcomas of female genital tract (35%), of non-visceral soft tissue and miscellaneous sites (32%) and of gastro-intestinal tract (13%). Numbers in the last category were, however, very small and the overall difference in survival by site was only of borderline significance ($P = 0.08$). The differences were less marked when relative survival was considered ($P = 0.2$). The survival of males and females with tumours of non-visceral soft tissue and miscellaneous sites was very similar ($P = 0.4$). Two of the five female patients with a tumour of the gastro-intestinal tract survived 5 years but all of the ten male patients died within 3 years ($P = 0.08$). Male patients with bone sarcomas had a marginally better survival than female patients but this difference did not attain statistical significance ($P = 0.3$).

Further sub-division of patients with tumours of non-visceral soft tissue into those with tumours of head, neck and trunk; extremities or retroperitoneum showed a statistically significant difference in survival between these sites ($P = 0.03$). This finding was related mainly to the very poor survival of patients with retroperitoneal tumours (5-year survival = 15%). There were no significant differences in survival by sex of patient for these three site sub-groups.

Stratification by age within the major sites indicated that crude survival appeared consistently better for the younger patients but the difference only attained statistical significance for non-visceral soft tissue combined with miscellaneous sites and for female genital tract. Five-year crude survival for the former group was 38% in patients aged under 65 years at diagnosis compared with 25% for those aged 65 years and over ($P = 0.04$). Difference in survival by

**Table III** Crude and relative five-year survival for reviewed bone and soft tissue sarcomas by diagnostic group and site

| Diagnostic group/site | No. in group | Median age at diagnosis (yrs) | Crude Survival: % survival at 5 years | Crude Survival: 95% CI | Relative Survival: % survival at 5 years | Relative Survival: 95% CI |
|---|---|---|---|---|---|---|
| All sarcomas | 310 | 59.5 (60)[a] | 34 | 29–39 | 40 | 34–46 |
| Males | 144 | 61 | 32 | 24–40 | 38 | 29–47 |
| Females | 166 | 57 | 36 | 29–43 | 41 | 32–50 |
| Aged < 60 | 155 | 39 | 43 | 35–51 | 44 | 36–52 |
| Aged ⩾ 60 | 155 | 71 | 24 | 17–31 | 34 | 25–43 |
| Soft tissue sarcomas | | | | | | |
| *Non-visceral soft tissue and miscellaneous sites* | 207 | 62 (65) | 32 | 26–38 | 39 | 31–47 |
| Males | 99 | 62 | 28 | 19–37 | 35 | 24–46 |
| Females | 108 | 61 | 36 | 27–45 | 43 | 32–54 |
| Aged < 65 | 117 | 50 | 38 | 29–47 | 40 | 31–49 |
| Aged ⩾ 65 | 90 | 73 | 25 | 16–34 | 37 | 23–51 |
| Head, neck & trunk | 87 | 61 (60) | 36 | 26–46 | 42 | 30–54 |
| Males | 37 | 62 | 35 | 20–51 | 43 | 24–62 |
| Females | 50 | 54 | 36 | 23–49 | 41 | 26–56 |
| Aged < 60 | 43 | 34 | 42 | 27–57 | 43 | 28–58 |
| Aged ⩾ 60 | 44 | 71 | 30 | 16–43 | 41 | 22–59 |
| Extremities | 82 | 67.5 (70) | 35 | 25–46 | 45 | 32–58 |
| Males | 42 | 66.5 | 31 | 17–45 | 39 | 22–57 |
| Females | 40 | 70.5 | 40 | 24–55 | 51 | 31–71 |
| Aged < 70 | 45 | 55 | 44 | 30–59 | 49 | 33–64 |
| Aged ⩾ 70 | 37 | 74 | 24 | 10–39 | 39 | 15–62 |
| Retroperitoneum | 27 | 58 (60) | 15 | 1–28 | 17 | 2–32 |
| *Female genital tract* | 31 | 54 (55) | 35 | 18–52 | 38 | 20–56 |
| Aged < 55 | 16 | 46.5 | 56 | 32–80 | 58 | 33–83 |
| Aged ⩾ 55 | 15 | 66 | 13 | 0–30 | 15 | 0–34 |
| *Gastro-intestinal tract* | 15 | 67 (70) | 13 | 0–30 | 17 | 0–39 |
| Leiomyosarcoma | | | | | | |
| Non-visceral soft tissue | 33 | 70 (70) | 21 | 6–36 | 27 | 8–46 |
| Female genital tract | 20 | 56 (55) | 25 | 6–44 | 27 | 6–48 |
| Gastro-intestinal tract | 14 | 69 (70) | 14 | 0–32 | 19 | 0–43 |
| MFH of soft tissue | 48 | 70.5 (70) | 29 | 16–42 | 39 | 22–56 |
| Sarcoma NOS of non-visceral soft tissue | 28 | 61.5 (60) | 32 | 15–49 | 38 | 18–58 |
| Liposarcoma | 21 | 59 (60) | 52 | 31–73 | 61 | 36–86 |
| All bone sarcomas | 57 | 33 (35) | 44 | 31–57 | 49 | 35–63 |
| Males | 35 | 33 | 51 | 35–67 | 59 | 40–78 |
| Females | 22 | 32.5 | 32 | 12–52 | 35 | 14–56 |
| Aged < 35 | 29 | 16 | 52 | 34–70 | 52 | 34–70 |
| Aged ⩾ 35 | 28 | 69 | 36 | 18–54 | 46 | 23–69 |
| Osteosarcoma of bone | 24 | 18 (20,30[b]) | 46 | 26–66 | 50 | 28–72 |
| Chondrosarcoma of bone | 22 | 63 (65) | 50 | 29–71 | 58 | 34–82 |

[a]Boundary used for comparison of survival by age group. [b]Including non-reviewed cases.

age for tumours of female genital tract was even more striking ($P = 0.005$, <55 yrs 56%, aged 55 + yrs 13%). Patients aged less than 35 years at diagnosis of their bone tumours had a higher 5-year survival rate than those over 35 years (52% *vs* 36%) but the difference was not significant ($P = 0.1$). Although the age differential in survival was maintained for each major site group when correction for underlying mortality was made, the only statistically significant difference remaining was that for tumours of female genital tract ($P = 0.01$).

Survival was also measured for certain diagnostic groups where numbers permitted, i.e. leiomyosarcoma, malignant fibrous histiocytoma (MFH), liposarcoma, osteosarcoma and chondrosarcoma.

Because a large proportion (38/71) of leiomyosarcomas in the series were of visceral sites, i.e. female genital tract and gastro-intestinal tract, and because leiomyosarcoma also formed the largest single group of tumours within these sites, separate survival curves were constructed to take site into account (Figure 4). Five-year survival was uniformly poor ranging from 14% for leiomyosarcoma of gastro-intestinal tract to 25% for that of female genital tract. Numbers in each sub-group, however, were small, and there was no significant difference overall ($P = 0.6$). Analyses by age and sex within each sub-group were generally inconclusive apart from a better relative survival for patients with leiomyosarcoma of female genital tract diagnosed under the median age of 55 years ($P = 0.05$, relative survival 45% *vs* 10%).

Survival in MFH was also low with only 29% of cases alive after 5 years, but survival in those aged under 70 years at diagnosis was strikingly better than in those aged 70 years and over (Figure 5), even when adjusted for underlying mortality ($P = 0.01$, relative survival overall 39%, <70 yrs 54%, 70 + yrs 20%).

Patients with liposarcoma had a 52% survival rate at 5 years and, as with MFH, there was a marked difference in survival by age group with older patients having a poorer prognosis (Figure 6). Overall relative survival at 5 years was

**Figure 1** Survival curves for all sarcoma patients by sex.

**Figure 2** Survival curves for all sarcoma patients by age at diagnosis.

**Figure 3** Survival curves for all sarcoma patients by site of tumour.

**Figure 4** Survival curves for patients with leiomyosarcoma by site of tumour.

**Figure 5** Survival curves for patients with malignant fibrous histiocytoma of soft tissue by age at diagnosis.

61%, 75% for those aged under 60 years at diagnosis and 41% for those aged 60 years and over ($P = 0.1$).

Survival rates for the two major groups of bone tumours, osteosarcoma and chondrosarcoma, were very similar with the greatest mortality in both occurring in the first 2–3 years. There was no significant difference for either sub-type by sex of patient but markedly different patterns emerged when the data were stratified by age at diagnosis, possibly because of the wide difference in the median age at diagnosis in the two groups. Osteosarcoma patients diagnosed under age 20 years had more than twice the 5-year survival rate than those diagnosed over this age ($P = 0.01$, 62% *vs* 27%) (Figure 7), a difference which was also apparent in relative survival rates ($P = 0.02$, 62% *vs* 34%). For patients with chondrosarcoma the difference in survival by age was not significant, but the numbers studied were small.

Even more striking differences in survival by age were seen for osteosarcoma when non-reviewed as well as reviewed cases were taken into consideration. Most of the non-reviewed cases of osteosarcoma (8/12) occurred in elderly individuals in whom diagnosis was based upon clinical criteria only. Inclusion of these in the analysis resulted in a

**Figure 6** Survival curves for patients with liposarcoma by age at diagnosis.

**Figure 8** Survival curves for patients with chondrosarcoma of bone by age at diagnosis.

higher median age at diagnosis for the group as a whole (29 years) and a much greater survival differential with 56% crude survival at 5 years for those aged less than 30 years at diagnosis and 11% for those aged 30 years and over ($P = 0.0004$). Relative survival was 56% *vs* 15% ($P = 0.002$).

### Discussion

Survival in sarcoma patients is difficult to assess reliably because of the rarity of such tumours, their differing topographical sites, the wide range of histological types seen and the difficulty of assessing degree of malignancy in certain cases. As a result of these factors, most published series which have included special pathology review have reported that a certain proportion of cases have been eliminated subsequent to re-classification as malignant tumours other than sarcomas, connective tissue tumours of borderline or uncertain malignant potential, benign tumours or non-neoplastic conditions (Presant *et al.*, 1986; Alvegård & Berg, 1989; El-Jabbour *et al.*, 1990, Agnarsson *et al.*, 1991). This is also the case in the present series where a non-sarcoma diagnosis was made in 22% of the cases originally entered in

**Figure 7** Survival curves for patients with osteosarcoma of bone by age at diagnosis.

the study (Harris *et al.*, 1991). Inclusion of these ineligible cases in any non-reviewed series may bias survival data.

Many studies have investigated prognostic variables for survival in sarcomas. Most of these have either concentrated upon features of the tumour itself, i.e. histological grade, size, depth, degree of necrosis etc. (Trojani *et al.*, 1984; Rööser *et al.*, 1988; Ueda *et al.*, 1988; Mandard *et al.*, 1989) or on various treatment modalities (Markhede *et al.*, 1982; Bramwell *et al.*, 1985; Suit *et al.*, 1985). In addition, most of these observations have been made on hospital-based or on selected sub-sets of population-based series.

Grading of tumours was not part of the protocol for this study so each group of sarcomas considered here is very mixed with respect to malignant potential. Nor was information on the presence of metastatic disease generally available at the time of case ascertainment. Hence the results represent a broad generalisation in terms of survival. In addition, because of the small numbers of some histological types the power of the study to detect differences in survival within and between groups was low in some cases.

Overall 5-year survival for the patients with reviewed histology entered in this series was 34%. In general, survival for bone sarcomas was marginally better than that for soft tissue tumours of any specified site i.e. bone 44%; female genital tract 35%; soft tissue sites 32%;and gastro-intestinal tract 13%, but this difference may be related to the younger age at diagnosis of the bone tumour patients. The prognosis for all the major histological groups of soft tissue tumours, other than for liposarcoma, was below the average for the entire series. The relatively good survival for liposarcoma probably reflects the inclusion of histological sub-types which are recognised to behave in a low grade manner, i.e. well differentiated and myxoid liposarcoma (Enzinger & Weiss, 1988).

Comparison with the population-based series of MFH reported from Sweden (Rööser *et al.*, 1991) indicated that survival in our series was much poorer (29% *vs* 72%) and that the very marked survival differential between younger and older patients was not apparent in the Swedish series. Possible reasons for these discrepancies could lie in the differing definitions of younger and older age groups for the two series, the higher median age at diagnosis in our series and the different proportions of histological sub-types within the two groups. No direct comparisons of survival by histological type with any other series is possible because of differences in ascertainment of patients and in the extent of centralised histopathology review.

Prognostic variables covered by this study other than site and histological type were age and sex. Although females overall had a slightly better 5-year survival rate than males (36% *vs* 32%), the difference was reversed for bone tumour patients (51% *vs* 32%) but neither difference was statistically significant. Better survival in female soft tissue sarcoma patients has been noted by Rööser *et al.* (1988), Ueda *et al.* (1988) and El-Jabbour *et al.* (1990), but no difference was apparent in other series (Bramwell *et al.*, 1985; Collin *et al.*, 1987).

The most consistent finding in these analyses was the better prognosis for younger patients as reported by other investigators (Bramwell *et al.*, 1985; Collin *et al.*, 1987; El-Jabbour *et al.*, 1990). Five year relative survival for patients who were under the median age at diagnosis of 60 years was 44% as opposed to 34% in those aged 60 years and over ($P = 0.03$). This survival differential was particularly striking for sarcomas (including leiomyosarcoma) of female genital tract, for MFH, osteosarcoma of bone and sarcoma NOS.

The wide divergence in survival between younger and older patients with osteosarcoma when all non-reviewed cases are taken into consideration is explained largely by the inclusion of a group of elderly patients, most of them male and with tumours associated with Paget's disease of bone in whom survival was particularly poor.

One of the encouraging results to emerge from the study was the high level of survival in children and young adults with osteosarcoma (62% at 5 years for patients aged under 20 years at diagnosis), a feature noted by Birch *et al.* (1988) for younger children registered with the Manchester Children's Tumour Registry, some of whom were also entered in this study. The improved level of survival for osteosarcoma in young people may be a reflection of the increasing trend towards the centralisation of treatment of childhood cancer in Britain which has been shown to have a favourable effect on survival for the period 1977–84 (Stiller, 1988). Most children and young adults in the North West region are treated centrally by consultants who are members of the United Kingdom Children's Cancer Study Group.

No analysis in general of survival by treatment centre was possible for patients in this series. Although a high proportion of patients was seen at the Christie Hospital and Holt Radium Institute, the major cancer treatment centre in the North Western region, no consistent information was available on reason for referral, i.e. for initial diagnosis, primary treatment, treatment of recurrence or palliation. Nor were treatment details available. Hence no comment can be made on how the survival patterns described here relate to primary treatment or adjuvant therapies.

The overall survival rates presented here may seem depressingly poor, especially those for the soft tissue tumours. It is important to bear in mind, however, that this series encompasses all cases from the North Western region, including those who were diagnosed at post mortem or died before treatment could be instituted, those who were considered too old or ill for active treatment, and those not referred to a primary treatment centre or referred only at the time of development of metastatic disease. Hence, although the study looked at a limited range of prognostic variables, i.e. histological type, site, age and sex, because it was truly population-based and included peer-review of histology, the results give a realistic assessment of overall survival for different types of sarcomas.

We are grateful to the North Western Regional Cancer Registry for the provision of data relating to registration of sarcomas and for data on cancer rates; to the North Western Regional Health Authority for population estimates; and to the Office of Population, Censuses and Surveys for mortality data (Crown copyright). We would also like to thank the many pathologists who provided material for the study including S. Banik, R.W. Blewitt, W.G. Brown, C.H. Buckley, J. Burns, A.B. Colclough, K.S. Daber, A.S. Day, D.M.H. De Krester, S. Dutt, A.R. Evans, G. Garrett, R. Gillett, J.R. Goepel, I. Gupta, B.N.A. Hamid, D.S. Harry, P.S. Hasleton, C.K. Heffernan, J.R. Helliwell, S.S. Hom-Choudhury, A.C. Hunt, N.N. Jaswon, A.R. Mainwaring, H.B. Marsden, J.A. Morris, H.M. Myat, W.G. Owen, N.L. Reeve, W.H. Richmond, C.M. Starkie, V. Tagore, W.H. Taylor, E.G.F. Tinsley, J.M. Torry, D.M. Vickers, S. Wells, J.S. Whittaker, G. Williams and H.D. Zakhour.

We should like to thank the staffs at the various medical records departments for their help in patient follow up, and the staff of the National Health Service Central Register for flagging and for provision of death notifications.

We are particularly grateful to Ewa Dale who scrutinised the cancer registrations and coordinated the receipt and dispatch of material, and to Joy Hogg who typed the manuscript.

This work was supported by the Cancer Research Campaign.

### References

AGNARSSON, B.A., BALDURSSON, G., BENEDIKTSDÓTTIR, K.R. & HRAFNKELSSON, J. (1991). Tumours in Iceland. 14. Malignant tumours of soft tissues. Histological classification and epidemiological considerations. *APMIS*, **99**,443–448.

ALVEGÅRD, T.A. & BERG, N.O. FOR THE SCANDINAVIAN SARCOMA GROUP (1989). Histopathology peer review of high-grade soft tissue sarcoma: The Scandinavian Sarcoma Group experience. *J. Clin. Oncol.*, **7**, 1845–1852.

BIRCH, J.M., MARSDEN, H.B., MORRIS JONES, P.H., PEARSON, D. & BLAIR, V. (1988). Improvements in survival from childhood cancer: results of a population based survey over 30 years. *Br. Med. J.*, **296**, 1372–1376.

BRAMWELL, V.H.C., CROWTHER, D., DEAKIN, D.P., SWINDELL, R. & HARRIS, M. (1985). Combined modality management of local and disseminated adult soft tissue sarcomas: A review of 257 cases seen over 10 years at the Christie Hospital and Holt Radium Institute, Manchester. *Br. J. Cancer*, **51**, 301–318.

BRAMWELL, V., QUIRT, I., WARR, D., VERMA, S., YOUNG, V., KNOWLING, M. & EISENHAUER. E, (1989). Combination chemotherapy with Doxorubicin, Dacarbazine, and Ifosfamide in advanced adult soft tissue sarcoma. *JNCI*, **81**, 1496–1499.

BUCKLEY, J.D. (1984). Additive and multiplicative models for relative survival rates. *Biometrics*, **40**, 51–62.

COLLIN, C., GODBOLD, J., HAJDU, S. & BRENNAN, M. (1987). Localized extremity soft tissue sarcoma: An analysis of factors affecting survival. *J. Clin. Oncol.*, **5**, 601–612.

CRAFT, A.W., AMINEDDINE, H.A., SCOTT, J.E.S. & WAGGET, J. (1987). The Northern region children's malignant disease registry 1968–82: Incidence and survival. *Br. J. Cancer*, **56**, 853–858.

EL-JABBOUR, J.N., AKHTAR, S.S., KERR, G.R., MCLAREN, K.M., SMYTH, J.F., RODGER, A. & LEONARD, R.C.F. (1990). Prognostic factors for survival in soft tissue sarcoma. *Br. J. Cancer*, **62**, 857–861.

EPILOG PLUS (1987). *Epicenter Software*. Pasadena: Epicenter Software.

ENZINGER, F.M. & WEISS, S.W. 2ND EDN (1988). *Soft Tissue Tumors*. C.V. Mosby Co. St. Louis.

FRAUMENI, J.F. & BOICE, J.D. (1982). Bone (review). In *Cancer Epidemiology and Prevention*. Schottenfeld, D. & Fraumeni, J.F. (eds) p. 814–826. W.B. Saunders: Philadelphia.

HARRIS, M., HARTLEY, A.L., BLAIR, V., BIRCH, J.M., BANERJEE, S.S., FREEMONT, A.J., MCCLURE, J. & MCWILLIAM, L.J. (1991). Sarcomas in North West England: I Histopathological Peer Review. *Br. J. Cancer*, **64**, 315–320.

HARTLEY, A.L., BLAIR, V., HARRIS, M., BIRCH, J.M., BANERJEE, S.S., FREEMONT, A.J., MCCLURE, J. & MCWILLIAM, L.J. (1991). Sarcomas in North West England: II Incidence. *Br. J. Cancer*, **64**, 1145–1150.

KAPLAN, E.L. & MEIER, P. (1958). Non-parametric estimation from incomplete observations. *J. Am. Stat. Assoc.*, **58**, 457–481.

MANDARD, A.M., PETIOT, J.C., MARNAY, J., MANDARD, J.C., CHASLE, J., DE RANIERI, E., DUPIN, P., HERLIN, P., DE RANIERI, J., TANGUY, A., BOULIER, N. & ABBATUCCI, J.S. (1989). Prognostic factors in soft tissue sarcomas. A multivariate analysis of 109 cases. *Cancer*, **63**, 1437–1451.

MARKHEDE, G., ANGERVALL, L. & STENER, B. (1982). A multivariate analysis of the prognosis after surgical treatment of malignant soft-tissue tumors. *Cancer*, **49**, 1721–1733.

PETO, R., PIKE, M.C., ARMITAGE, P., BRESLOW, N.E., COX, D.R., HOWARD, S.V., MANTEL, N., MCPHERSON, K., PETO, J. & SMITH, P.G. (1977). Design and analysis of randomised clinical trials requiring prolonged observation of each patient. II Analysis and examples. *Br. J. Cancer*, **35**, 1–39.

PRESANT, C.A., RUSSELL, W.O., ALEXANDER, R.W. & FU, Y.S. (1986). Soft tissue and bone sarcoma histopathology peer review: The frequency of disagreement in diagnosis and the need for second pathology opinions. The Southeastern Cancer Study Group experience. *J. Clin. Oncol.*, **4**, 1658–1661.

RÖÖSER, B., ATTEWELL, R., BERG, N.O. & RYDHOLM, A. (1988). Prognostication in soft tissue sarcoma. A model with four risk factors. *Cancer*, **61**, 817–823.

RÖÖSER, B., WILLÉN, H., GUSTAFSON, P., ALVEGÅRD, T.A. & RYDHOLM, A. (1991). Malignant fibrous histiocytoma of soft tissue. A population-based epidemiologic and prognostic study of 137 patients. *Cancer*, **67**, 499–505.

STILLER, C.A. (1988). Centralization of treatment and survival rates for cancer. *Arch. Dis. Child.*, **63**, 23–30.

STOTTER, A.T., A'HERN, R.P., FISHER, C., MOTT, A.F., FALLOWFIELD, M.E. & WESTBURY, G. (1990). The influence of local recurrence of extremity soft tissue sarcoma on metastasis and survival. *Cancer*, **65**, 1119–1129.

SUIT, H.D., MANKIN, H.J., WOOD, W.G. & PROPPE, K.H. (1985). Preoperative, intraoperative and postoperative radiation in the treatment of primary soft tissue sarcoma. *Cancer*, **55**, 2659–2667.

TROJANI, M., CONTESSO, G., COINDRE, J.M., ROUESSE, J., BUI, N.B., DE MASCAREL, A., GOUSSOT, J.F., DAVID, M., BONICHON, F. & LAGARDE, C. (1984). Soft-tissue sarcomas of adults; study of pathological prognostic variables and definition of a histopathological grading system. *Int. J. Cancer*, **33**, 37–42.

TUCKER, M.A. & FRAUMENI, J.F. (1982). Soft Tissue (review). In *Cancer Epidemiology and Prevention*. Schottenfeld, D & Fraumeni, J.F. (eds) p. 827–836. W.B. Saunders: Philadelphia.

UEDA, T., AOZASA, K., TSUJIMOTO, M., HAMADA, H., HAYASHI, H., ONO, K. & MATSUMOTO, K. (1988). Multivariate analysis for clinical prognostic factors in 163 patients with soft tissue sarcoma. *Cancer*, **62**, 1444–1450.

WORLD HEALTH ORGANISATION (1976). ICD-0:*International Classification of Diseases for Oncology*. World Health Organisation, Geneva.